# Complement Dysregulation in Obese Versus Nonobese Polycystic Ovary Syndrome Patients

**DOI:** 10.3390/cells12152002

**Published:** 2023-08-04

**Authors:** Alexandra E. Butler, Abu Saleh Md Moin, Thozhukat Sathyapalan, Stephen L. Atkin

**Affiliations:** 1 Royal College of Surgeons in Ireland Bahrain, Busaiteen P.O. Box 15503, Adliya, Bahrain; amoin@rcsi.com (A.S.M.M.); satkin@rcsi.com (S.L.A.); 2 Academic Endocrinology, Diabetes and Metabolism, Hull York Medical School, Hull HU6 7RU, UK; thozhukat.sathyapalan@hyms.ac.uk

**Keywords:** polycystic ovary syndrome, complement factors, factor H, C3, properdin, factor B

## Abstract

Introduction: Upregulation of complement system factors are reported to be increased in polycystic ovary syndrome (PCOS) and may be due to obesity and insulin resistance rather than inherently due to PCOS. We directly compared complement factors from an obese, insulin-resistant PCOS population to a nonobese, non-insulin-resistant PCOS population in a proteomic analysis to investigate this. Methods: Plasma was collected from 234 women (137 with PCOS and 97 controls) from a biobank cohort and compared to a nonobese, non-insulin-resistant population (24 with PCOS and 24 controls). Slow off-rate modified aptamer (SOMA) scan plasma protein measurement was undertaken for the following complement system proteins: C1q, C1r, C2, C3, C3a, iC3b, C3b, C3d, C3adesArg, C4, C4a, C4b, C5, C5a, C5b-6 complex, C8, properdin, factor B, factor D, factor H, factor I, Mannose-binding protein C (MBL), complement decay-accelerating factor (DAF) and complement factor H-related protein 5 (CFHR5). Results: The alternative pathway of the complement system was overexpressed in both obese and nonobese PCOS, with increased C3 (*p* < 0.05) and properdin (*p* < 0.01); additionally, factor B increased in obese PCOS (*p* < 0.01). For inhibitors of this pathway, factor I was increased (*p* < 0.01) in both slim and obese PCOS, with an increase in CFHR5 and factor H in obese PCOS (*p* < 0.01). Complement factors iC3b, C3d and C5a, associated with an enhanced B cell response and inflammatory cytokine release, were increased in both slim and obese PCOS (*p* < 0.05). C3a and its product, C3adesArg, were both significantly elevated in nonobese PCOS (<0.01) but not altered in obese PCOS. Hyperandrogenemia correlated positively with properdin and iC3b in obese PCOS (*p* < 0.05) but not in nonobese PCOS. There was no association with insulin resistance. BMI correlated positively in both groups with factor B, factor H and C5a. Additionally, in obese PCOS, BMI correlated with C3d, factor D, factor I, CFHR5 and C5a (*p* < 0.05), and in nonobese PCOS, BMI correlated with properdin, iC3b, C3, C3adesArg, C3a, C4, C5, C5a and C1q. In obese controls, BMI correlated with C3, C3desArg, C3a, C3d, C4, factor I, factor B, C5a and C5, whilst in nonobese controls, BMI only correlated negatively with C1q. Comparison of nonobese and obese PCOS showed that properdin, C3b, iC3b, C4A, factor D, factor H and MBL differed. Conclusion: The upregulation of the alternative complement pathway was seen in nonobese PCOS and was further exacerbated in obese PCOS, indicating that this is an inherent feature of the pathophysiology of PCOS that is worsened by obesity and is reflected in the differences between the nonobese and obese PCOS phenotypes. However, the increase in the complement proteins associated with activation was counterbalanced by upregulation of complement inhibitors; this was evident in both PCOS groups, suggesting that insults, such as a cardiovascular event or infection, that cause activation of complement pathways may be amplified in PCOS.

## 1. Introduction

**Figure 1:**
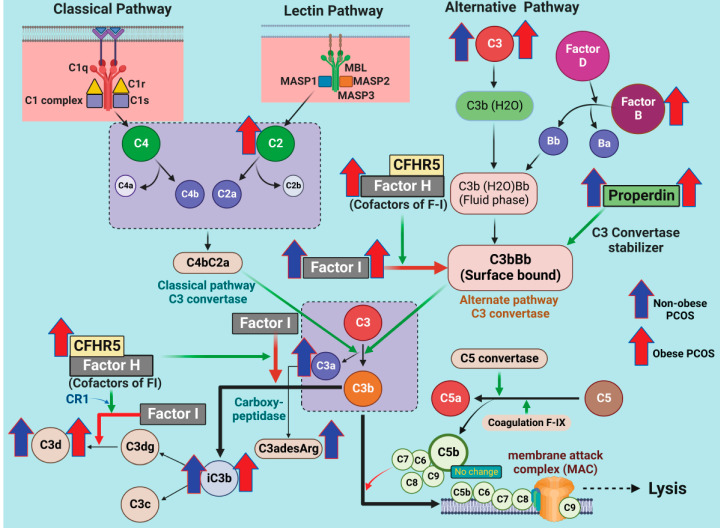
A schematic to illustrate the proteins involved in the three arms of the complement cascade (classical, alternative and lectin pathways). Thick blue arrows (pointing upwards) show the complement proteins found to be increased in nonobese PCOS women. Thick red arrows (pointing upwards) show complement proteins found to be increased in obese PCOS women. Green arrows show enzyme activity or positive regulation, whilst red arrows show pathway inhibition. The illustration was created using BioRender.com (with publication license).

Whilst it is recognized that polycystic ovary syndrome (PCOS) is the most common endocrine disorder in premenopausal women leading to anovulatory infertility, the mechanisms causing the metabolic dysregulation leading to an increased prevalence of type 2 diabetes, hypertension and, potentially, cardiovascular disease [1], remain unclear. Inflammation, directly through the inherent PCOS phenotype or indirectly through obesity and insulin resistance, has been suggested as the main mediator for PCOS pathophysiology [2], though other mechanisms involving oxidative stress mediated through hyperandrogenemia, vitamin D levels and insulin resistance have also been suggested [3,4]. The enhanced inflammation and oxidative stress are associated with other cellular markers such as dysregulation of heat shock proteins [5], alteration in coagulation markers [6] and alteration in complement pathway proteins in which obesity and insulin resistance have been suggested to be the underlying mediators [7,8,9,10]. Classical and alternate complement pathways have been implicated, with elevation in C3, C4, properdin, factor B and factor D [7,10] having been reported in PCOS (Figure 1), though their expression was correlated to obesity and insulin resistance [7]; however, there are conflicting reports on complement proteins, suggesting that C3 may be elevated and related to inflammation [8,9,10] whilst others report that C3 levels are unchanged [11]. One study reported that C3 was higher in PCOS compared to controls at baseline but was unaffected following 3 months of exercise and was unrelated to insulin resistance (that was reduced in response to exercise) [12].

Classical, alternate and lectin pathways activate the complement system that is involved in innate and adaptive immunity and that is integral to the inflammatory response [13]; these three pathways lead to a common pathway as shown in Figure 1. Complement activation has been shown to be involved in several disease processes that are associated with PCOS, including diabetes (and diabetes-related complications) [14] and cardiovascular disease [15].

The issues complicating the analysis of complement proteins in PCOS are that the obesity and insulin resistance with accompanying inflammation are so highly correlated to PCOS that statistical regression adjustment for BMI over-adjusts PCOS effects; therefore, in this study, a comparison between obese PCOS and PCOS subjects who were nonobese, non-insulin-resistant, and without increased systemic inflammation was undertaken using a comprehensive panel of complement proteins to determine whether the complement factor proteins are independently associated with PCOS. 

## 2. Materials and Methods

### 2.1. Obese PCOS and Controls Cohort [10]

We determined plasma levels of classical and alternative complement pathway proteins in PCOS (*n* = 137) and control (*n* = 97) women recruited to a PCOS biobank (ISRCTN70196169). All patients provided written informed consent. Clinical data and samples were accessed from the PCOS Genetic Biobank in the UK, with approval from the Newcastle & North Tyneside ethics committee. 

All study subjects were ethnically Caucasian. To reach a diagnosis of PCOS, at least two of three diagnostic criteria as outlined by the Rotterdam consensus had to be satisfied [16]; these specific criteria are clinical plus biochemical hyperandrogenism (indicated by a Ferriman–Gallwey score of 8 or greater, a free androgen index (FAI) of 4 or greater, a total testosterone level of 1.5 nmol/L or greater), oligomenorrhea or amenorrhea together with polycystic ovaries as assessed using transvaginal ultrasound (TVUS). The following endocrine conditions were ruled out by performing appropriate testing: nonclassical 21-hydroxylase deficiency, hyperprolactinemia, Cushing’s disease and androgen-secreting tumors. All the obese PCOS cohort fulfilled all three of the Rotterdam criteria (phenotype A) whilst, in the nonobese PCOS cohort, 10 were phenotype A and 14 were phenotype B (irregular periods and hyperandrogenemia). Baseline data have previously been detailed [17] and Table 1 depicts demographic data for both cohorts (PCOS and control) of women. Importantly, control women (who had been recruited for the study using adverts) all reported regular periods, and had no evidence of clinical/ biochemical hyperandrogenism or polycystic ovaries by TVUS; they neither had any significant medical history nor were taking medication of any kind (including oral contraceptive pills or over-the-counter medication).

### 2.2. Nonobese PCOS and Control Cohort [9]

We determined plasma complement pathway protein levels in sequential women with PCOS (*n* = 24) and control (*n* = 24) women attending the Hull IVF clinic [18] and prior to any IVF therapy. All PCOS women presented with anovulatory infertility. Control women were age- and BMI-matched to the women with PCOS. All subject inclusion criteria were age 20–40 years, BMI ≤ 30; control women’s reason for in vitro fertilization was either unexplained infertility (*n* = 5) or male factor infertility (*n* = 19). The World Health Organization (WHO) definition of obesity, a BMI greater or equal to 30 kg/m^2^, was used in this study [19]. All subjects fulfilling the inclusion and exclusion criteria who were attending for a routine clinical mock embryo transfer procedure were approached about inclusion in the study and signed written informed consent. Study approval was granted by The Yorkshire and The Humber NRES ethical committee, UK (approval number 02/03/043).

### 2.3. Sample Analysis

Blood was drawn when the subjects were in a fasting state. Immediately thereafter, it was centrifuged (3500× *g*, 15min), aliquoted and frozen at −80 °C in preparation for analysis. Analysis was performed for the following parameters: insulin and sex hormone-binding globulin (SHBG), (DPC Immulite 200 analyzer, Euro/DPC, Llanberis, UK), glucose (plasma; Synchron LX20 analyzer, Beckman-Coulter, High Wycombe, UK). Free androgen index (FAI) was determined by dividing total testosterone by SHBG and multiplying by 100. Insulin resistance (IR) was determined using the homeostasis model assessment (HOMA-IR). Testosterone levels in serum were determined using isotope dilution liquid chromatography–tandem mass spectrometry (LC-MS/MS) [18]. 

Complement pathway protein levels were measured in plasma using slow off-rate modified aptamer (SOMA) scan (SomaLogic, Boulder, CO, USA), the methodology of which has been previously detailed [20], and followed the standard protocol (1. normalization of raw intensities; 2. hybridization; 3. median signal and calibration signal determination based upon standard samples incorporated onto each plate) [21]. 

SOMAscan assay v3.1 of the, targeting complement pathway proteins was undertaken; some of these proteins have previously been reported by others [7]. The specific list of proteins targeted is as follows: Complement factor H-related protein 5 (CFHR5), properdin, MBL, complement decay-accelerating factor (DAF), C1r, C1q, C2, C3, C3a, C3b, iC3b, C3adesArg, C3d, C4, C4a, C4b, C5, C5a, C5b-6 complex, C8, factors B, D, H and I, (Figure 1, Table 2).

### 2.4. Statistics

A power analysis (nQuery version 9, Statsol, San Diego, CA, USA) was undertaken for the C3 protein previously reported to be different in PCOS [7]. To achieve 80% power with alpha 0.05 and common standard deviation 0.37, the required number of participants was determined to be *n* = 23. Data were evaluated for normality both visually and statistically. Where there was a normal distribution, a Student *t*-test was used; if not normally distributed, as determined using the Kolmogorov–Smirnov test, the nonparametric Mann–Whitney U test was used. Correlation analyses between the complement proteins and BMI were performed with Pearson coefficient. All analyses were performed using R version 4.0.0 (R Foundation for Statistical Computing, Vienna, Austria. URL https://www.R-project.org/).

## 3. Results

Baseline data for the 146 PCOS patients and 97 controls and for the nonobese, non-insulin-resistant PCOS (*n* = 24) and controls (*n* = 24) are shown in Table 1. For the obese cohort, age was matched, but PCOS subjects had a greater BMI, showed increased insulin resistance, hyperandrogenemia and increased CRP (as a marker of inflammation). For the nonobese, non-insulin-resistant cohort, age and BMI were matched, and the women with PCOS were not insulin-resistant, and nor was CRP elevated. 

The results of the complement factors are shown in Table 2 for the obese and nonobese PCOS and their respective control subjects, and between the nonobese and obese PCOS cohorts.

### 3.1. Alternative Pathway (AP) Proteins of Complement Activation in PCOS

The levels of alternative pathway complement activation component C3 were higher in both obese PCOS (*p* = 0.04, obese PCOS vs. control) and nonobese PCOS (*p* = 0.002, non-obese PCOS vs. control); however, the functional fragment of C3 cleavage, C3a, was only elevated in nonobese PCOS compared to controls (*p* = 0.007), together with the elevation of its product C3desArg (*p* = 0.004), but was no different in obese PCOS. The functional fragment of C3 cleavage, C3b, was not different in obese PCOS or nonobese PCOS women compared to their controls (Table 2); however, its cleavage product iC3b was significantly elevated in both nonobese PCOS (*p* = 0.02) and obese PCOS (*p* < 0.001) compared to controls. Among the positive regulators of the alternative pathway, factor B (F-B) was higher in obese PCOS (*p* < 0.0001, obese PCOS vs. control), but did not differ in nonobese PCOS compared to controls (Table 2); however, factor D (F-D) did not differ in either obese or nonobese PCOS compared to their respective controls. The levels of properdin were higher in both obese PCOS (*p* < 0.0001, obese PCOS vs. control) and nonobese PCOS (*p* = 0.006, nonobese PCOS vs. control). Among the negative regulators of the alternative pathway, the levels of factor H (F-H) were significantly elevated in obese PCOS (*p* < 0.0001, obese PCOS vs. control) and factor I (F-I) was higher in both nonobese PCOS (*p* = 0.01, nonobese PCOS vs. control) and obese PCOS (*p* < 0.0001, obese PCOS vs. control) compared to controls. Complement factor H-related protein (CFHR5) levels were higher in obese PCOS (*p* = 0.01, obese PCOS vs. control) but did not differ in nonobese PCOS versus controls. The levels of the degradation products (by F-I) iC3b (*p* < 0.001, obese PCOS vs. control; *p* < 0.02, nonobese PCOS vs. control) and C3d (*p* < 0.0001, obese PCOS vs. control; *p* = 0.02, nonobese PCOS vs. control) were also higher in both obese and nonobese PCOS compared to their controls.

Comparison of nonobese and obese PCOS showed that properdin, C3b, iC3b, C4A, factor D, factor H and MBL differed significantly between the cohorts following Bonferroni correction for multiple comparisons.

### 3.2. Lectin Pathway (LP) Proteins of Complement Activation in PCOS

The levels of C2, a major component of the lectin pathway of complement activation, was higher in obese PCOS compared to control (*p* = 0.002, obese PCOS vs. control), but did not differ in nonobese PCOS compared to controls. There was no difference in MBL for obese and nonobese PCOS and their respective controls.

### 3.3. Classical Pathway (CP) Proteins of Complement Activation in PCOS

There was no change in classical pathway components C1q and C1r levels in either obese PCOS or nonobese PCOS vs. their controls (Table 2). C4a and C4b did not differ between groups, nor were there any differences in C5 or C5b-6 complex (Table 2). 

### 3.4. Correlations of Complement Activation-Related Proteins with Hyperandrogenemia and BMI

Hyperandrogenemia correlated positively with properdin (*r* = 0.19, *p* = 0.047) and iC3b (*r* = 0.31, *p* = 0.001) in obese PCOS but hyperandrogenemia did not correlate with any complement factors in nonobese PCOS.

### 3.5. Correlations of Complement Activation in Obese and Nonobese PCOS with BMI

**Figure 2:**
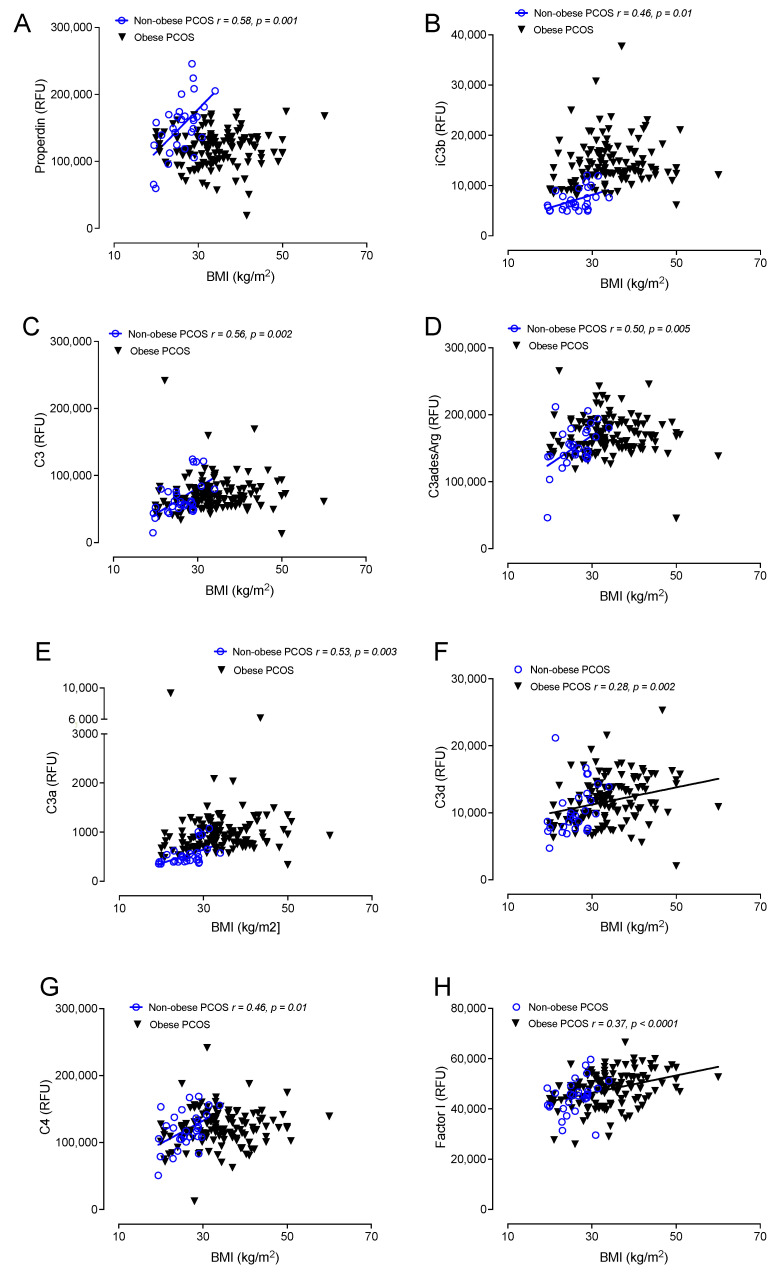
Correlations of BMI with complement pathway proteins in obese patients with PCOS (*n* = 137) and in nonobese PCOS patients (*n* = 24). Correlations of properdin (**A**), iC3b (**B**), C3 (**C**), C3adesArg (**D**), C3a (**E**), C3d (**F**), C4 (**G**) and factor I (**H**) with BMI. Relative fluorescent units (RFU).

**Figure 3:**
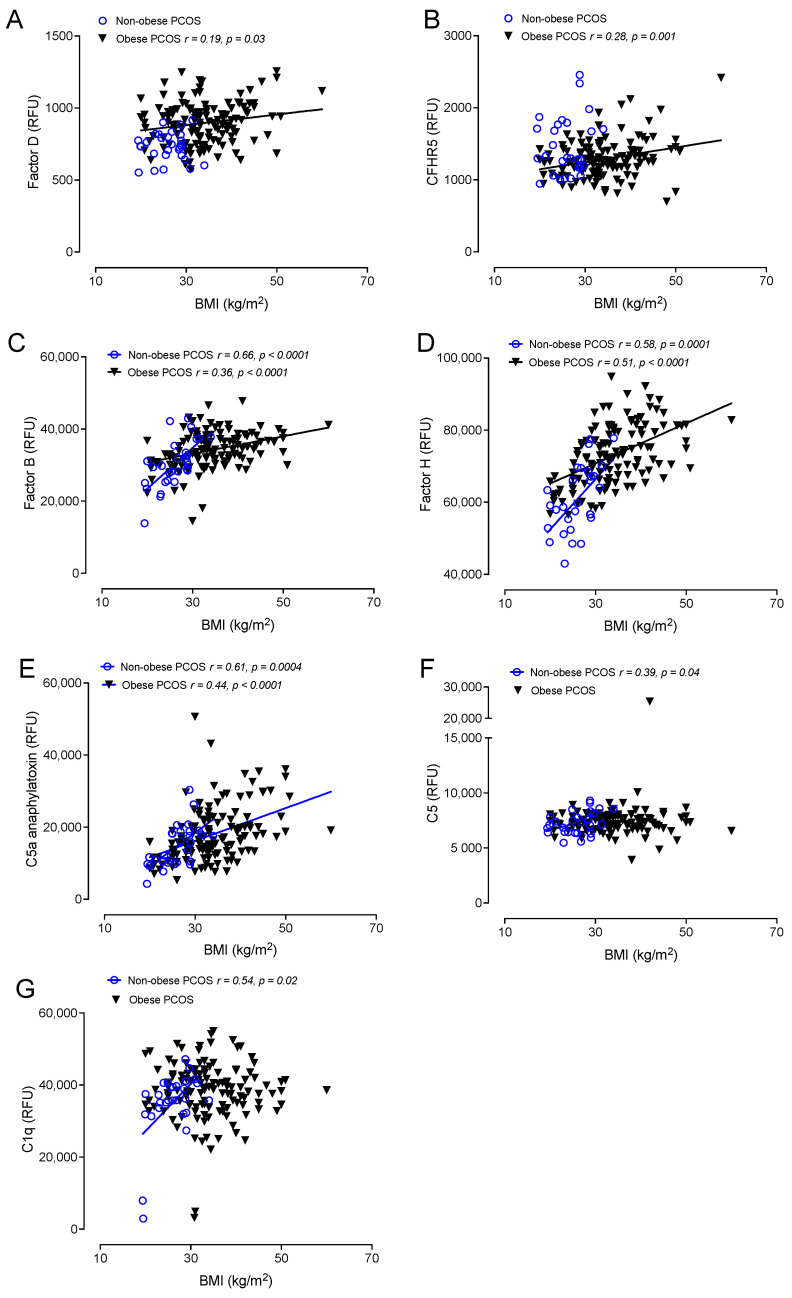
Correlations of BMI with complement pathway proteins in obese patients with PCOS (*n* = 137) and in nonobese PCOS patients (*n* = 24). Correlations of factor D (**A**), CFHR5 (**B**), factor B (**C**), factor H (**D**), C5a anaphylatoxin (**E**), C5 (**F**) and C1q (**G**) with BMI. Relative fluorescent units (RFU).

Correlations between obese and nonobese PCOS with BMI are shown in Figure 2 and Figure 3. BMI correlated positively in both obese and nonobese PCOS with factor B (*r* = 0.36, *p* < 0.0001 obese PCOS: *r* = 0.66, *p* < 0.0001 nonobese PCOS), factor H (*r* = 0.51, *p* < 0.0001 obese PCOS: *r* = 0.58, *p* < 0.0001 nonobese PCOS) and C5a (*r* = 0.44, *p* < 0.0001 obese PCOS: *r* = 0.61, *p* < 0.0004 nonobese PCOS). 

In obese PCOS, BMI additionally correlated with C3d (*r* = 0.28, *p* = 0.002), factor I (*r* = 0.37, *p* < 0.0001), factor D (*r* = 0.19, *p* = 0.03) and CFHR5 (*r* = 0.28, *p* = 0.001). 

In nonobese PCOS, BMI additionally correlated with properdin (*r* = 0.58, *p* = 0.001), iC3b (*r* = 0.46, *p* = 0.01), C3 (*r* = 0.56, *p* = 0.002), C3adesArg (*r* = 0.5, *p* = 0.001), C3a (*r* = 0.53, *p* = 0.003), C4 (*r* = 0.46, *p* = 0.01), C5 (*r* = 0.39, *p* = 0.04) and C1q (*r* = 0.54, *p* = 0.02).

### 3.6. Correlations of Complement Activation in Obese and Nonobese Control Subjects with BMI

**Figure 4:**
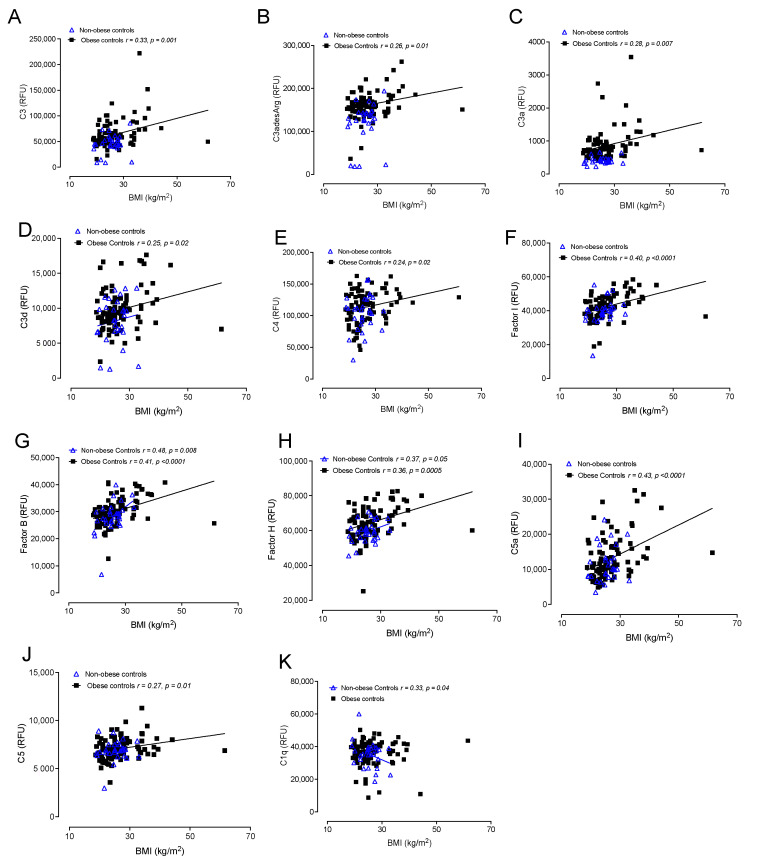
Correlations of BMI with complement pathway proteins in obese controls (*n* = 97) and in nonobese control subjects (*n* = 24). Correlations of C3 (**A**), C3adesArg (**B**), C3a (**C**), C3d (**D**), C4 (**E**), factor I (**F**), factor B (**G**), factor H (**H**), C5a (**I**), C5 (**J**) and C1q (**K**) with BMI. Relative fluorescent units (RFU).

Correlations between obese and nonobese controls with BMI are shown in Figure 4. In obese controls, positive correlations with BMI were found for C3d (*r* = 0.25, *p* = 0.02), factor I (*r* = 0.40, *p* < 0.0001), factor B (*r* = 0.41, *p* < 0.0001), factor H (*r* = 0.36, *p* = 0.0005), C5a (*r* = 0.43, *p* < 0.0001), iC3b (*r* = 0.24, *p* = 0.02), C3 (*r* = 0.33, *p* = 0.001), C3adesArg (*r* = 0.26, *p* = 0.01), C3a (*r* = 0.28, *p* = 0.007), C4 (*r* = 0.24, *p* = 0.02) and C5 (*r* = 0.27, *p* = 0.01), whilst in nonobese control, BMI only correlated negatively with C1q (*r*= −0.33, *p* = 0.04).

## 4. Discussion

In this study, we used a PCOS comparator group that was nonobese, non-insulin-resistant and without increased systemic inflammation to address and circumvent the effects of obesity, insulin resistance and inflammation, which are difficult to account for statistically without undermining the effects seen between PCOS subjects and controls. Here, we report the complement activation system and their correlations with hormonal and metabolic parameters in women with obese and nonobese PCOS. The data show that the alternative pathway proteins of the complement system were overexpressed in both obese and nonobese cohorts for PCOS, with increased C3 and properdin, though factor B (F-B) was only increased in obese PCOS, which correlated with BMI for both the obese and nonobese cohorts. This suggests that C3 and properdin are inherently increased in the PCOS phenotype independent of BMI, insulin resistance and inflammation, whilst F-B is obesity-dependent and hence only differs in obese PCOS. The increases in factors associated with alternate pathway activation appeared to be balanced by an increase in the inhibitory factor I (F-I) in both obese and nonobese PCOS, which also correlated with BMI in obese PCOS, suggesting that F-I is inherently increased in the PCOS phenotype, but that it is further exacerbated by obesity. Other alternate pathway inhibitory proteins, CFHR5 and factor H (F-H), were only increased in obese PCOS and both correlated with BMI, suggesting that obesity was causing their elevation; this is also perhaps an indication that in obese PCOS there is exacerbated alternate pathway activation, requiring more factors to damp it down. In addition, iC3b, which prevents amplification of the alternative pathway cascade, as it cannot associate with F-B [22], was increased in both obese and nonobese PCOS women, thereby inhibiting the alternative pathway. It should also be noted that hyperandrogenism was present in both obese and nonobese PCOS but correlated with properdin and iC3b in obese PCOS alone, suggesting that hyperandrogenism per se has little effect on the complement system and any effects may be an epiphenomenon of obesity. In this comparison between the obese and nonobese PCOS cohorts, there was no difference in key recognition molecules of the classical pathway (C1r, C1q) or the lectin pathway (MBL) of complement activation in PCOS subjects. However, there was an increase in C2, associated with lectin pathway activation, in obese PCOS that was not seen in the nonobese cohort and that did not associate with BMI, hyperandrogenism or insulin resistance, suggesting that an additional unaccounted for factor may be involved.

The comparison of nonobese and obese PCOS showed that properdin, C3b, iC3b, C4A, factor D, factor H and MBL differed. Of the complement proteins that differed for both obese and nonobese PCOS compared to controls, only properdin differed significantly whilst C3, C3d, factor I and C5a did not, suggesting that the magnitude of the differences in both obese and nonobese PCOS were the same. However, where there were no differences between PCOS and controls, there were differences seen between the two PCOS cohorts for C4A, factor D, factor H and MBL; these would, however, likely be accounted for by BMI differences, as seen in the correlation analyses.

Complement activation is triggered by target recognition and the data here suggest that the pattern recognition molecules for both classical pathway (C1q and C1r) and lectin pathway (MBL) activation differed for neither the obese nor nonobese cohorts, indicating that in normal homeostasis the alternative pathway is kept in check by the inhibiting proteins and the classical and lectin pathways of complement activation are not activated in PCOS.

The main active complement pathway is the alternative pathway (AP) that both monitors for foreign pathogen invasion or eliminates dying host cells by maintaining low-level constitutive activation. Each of the three pathways results in the activation of the C3 protein by cleavage into functional active fragments, C3a and C3b [23]. C3a acts as an inflammatory mediator and was elevated in both obese and nonobese PCOS, whilst C3b did not differ in either obese or nonobese PCOS; however, its cleavage product iC3b, which prevents amplification of the alternative pathway [22], was increased in both obese and nonobese PCOS, thereby inhibiting the alternative pathway. C3a is cleaved to C3adesArg and most of the plasma C3a is in the C3adesArg form; C3adesArg was elevated in nonobese PCOS but not obese PCOS, which appears counterintuitive, as C3adesArg is strongly associated with insulin resistance and inflammation [24] that was seen in the obese PCOS cohort.

The effect of BMI on the complement proteins, independent of PCOS, was seen in the control cohorts. In the obese control women, BMI correlated with C3, C3desArg, C3a, C3d, C4, factor I, factor B, C5a and C5 whilst, in the nonobese control women, BMI only correlated negatively with C1q, again suggesting that the complement protein changes are due to a combination of those inherent in the pathophysiology of PCOS as well as due to additional physiological factors such as obesity.

Factor H is the soluble inhibitor of C3 convertase that competes with F-B for binding to C3b to regulate the AP and the amplification loop of the complement pathway [25]. Factor H was significantly increased in obese PCOS but not in the nonobese PCOS cohort and was significantly associated in both cohorts with BMI; likewise, it was associated with BMI in the obese control cohort, suggesting that obesity is the major mechanistic factor in its elevation [7]. Factor I (F-I) cleaves C3b, leading to the degradation products C3b and iC3b, which is then unable to bind F-B [26]; F-I was elevated in both obese and nonobese PCOS and associated with BMI in obese PCOS, suggesting contributions in its elevation from both the inherent PCOS phenotype and obesity. Therefore, elevation of both inhibitors in PCOS likely reflects dysregulation of the AP in PCOS. DAF was unchanged in both the obese and nonobese PCOS compared to their respective controls and between the obese and nonobese PCOS cohorts, which is surprising, as it acts to accelerate the decay in the classical and alternative C3 and C5 convertases [27] and, as is shown here, there were changes in C3 and C5.

Complement factor H-related protein 5 (CFHR5) levels were elevated only in obese PCOS compared to controls and were associated with BMI in obese PCOS, suggesting that obesity is responsible for the changes seen. CFHR5 is reported to have complement regulatory activities as it binds to heparin and C3b and functions as a cofactor of F-I in C3b cleavage [28], further supporting the concept of hyperactivation of regulatory proteins of the AP in PCOS.

In the terminal complement pathway, C5 convertase cleaves an inert molecule of C5 into a potent anaphylatoxin, C5a, and a bioactive fragment C5b (Figure 1). Whilst C5 did not differ between obese and nonobese PCOS cohorts, C5a was elevated in both obese and nonobese PCOS cohorts and associated with BMI in the nonobese PCOS cohort and in controls in the obese PCOS cohort. Thus, it remains unclear if these changes are inherently due to PCOS or not. C5 and C5b, 6 complex did not differ in PCOS, potentially suggesting the unavailability of the initiating components (C5b, which upon cleavage undergoes a conformational change to interact with C6) of membrane attack complex (MAC) formation in the PCOS women. It has been previously reported that coagulation/fibrinolysis proteases (thrombin, coagulation factors IX, XI, X and plasmin) may act as natural C3 and C5 convertases [29], and coagulation factor IX is significantly higher in PCOS, suggesting that factor IX might play a role in the elevation of C5a in patients with PCOS.

Clearly, on a day-to-day basis, the complement system in women with PCOS is in homeostasis, but, hypothetically, a superimposed event leading to complement activation, such as an infection, may lead to a worse outcome for both obese and nonobese PCOS women due to an exaggerated complement response [30], and there is indeed evidence for this [31,32].

In general, few studies have been performed in the nonobese PCOS population and fewer still focused upon the complement system/immune system. One such study in a nonobese PCOS population showed lower levels of IgG free light chains and higher hemolytic complement activity, indicative of the involvement of mechanisms other than the “classical” pathway of complement activation [33]. That report is in accordance with the findings in this study, though the nonobese PCOS differed in that chronic inflammation was present in that cohort [33] but not in the cohort reported here.

A strength of this study is that the same proteomic platform was used for both of the cohort studies, but a limitation is that the protein panel analysis for each study was performed separately and not as part of a single study; therefore, absolute changes between the PCOS groups could not be undertaken to account for assay differences. A further limitation is that only circulatory protein levels were measured, and no functional studies were undertaken to confirm that the alternative complement system was in balance, and if this balance was disrupted, would it result in an enhanced complement response in the setting of PCOS. In the obese PCOS cohort, the control population differed in BMI, and it would have been better to match for BMI in this cohort also to avoid any potential biases in the results. PCOS phenotype may be important in the complement system [33,34] and needs to be investigated in the presence and absence of obesity and insulin resistance. In this study, the obese PCOS group fulfilled all three of the Rotterdam criteria and thus were phenotype A. There were too few of phenotype A versus B in the nonobese PCOS cohort to perform a robust statistical analysis on the complement proteins; therefore, future studies should address the impact of the differing phenotypes according to the Rotterdam criteria on the complement system in PCOS [33]. The WHO definition of obesity was used in this study; it would be of value to undertake body composition measurements and compare those to the complement proteins, though the relationship of body composition to the pathophysiology of PCOS suggests that global obesity burden rather than regional fat distribution is the most important factor in PCOS [35]; thus, body composition may not affect the complement system.

In conclusion, the upregulation of the alternative complement pathway was seen in nonobese PCOS and was further exacerbated in obese PCOS, indicating that this is an inherent feature of the pathophysiology of PCOS that is worsened by obesity and is reflected in the differences between the nonobese and obese PCOS phenotypes. However, the increase in the complement proteins associated with activation was counterbalanced by upregulation of complement inhibitors; this was evident in both PCOS groups, suggesting that insults—such as a cardiovascular event or infection—that cause activation of complement pathways may be amplified in PCOS.

## Figures and Tables

**Table 1:** Demographics, baseline hormonal and metabolic parameters of the obese and lean polycystic ovary syndrome (PCOS) subjects and their respective controls.

Baseline Demographics	PCOS Obese (*n* = 137)	Control Lean (*n* = 97)	*p* Value	PCOS Lean (*n* = 24)	Control Lean (*n* = 24)	*p* Value
	Mean (SD)	Mean (SD)		Mean (SD)	Mean (SD)	
Age (years)	29.1 ± 6.1	29.6 ± 6.5	0.09	31 ± 6.4	32.5 ± 4.1	0.09
BMI (kg/m²)	34.1 ± 7.5	26.7 ± 6.6	<0.0001	25.9 ± 1.8	24.8 ± 1.1	0.44
Insulin (IU/mL)	10.2 ± 6.1	6.2 ± 3.2	0.001	8.1 ± 4.7	7.7 ± 4.0	0.66
HOMA-IR	3.8 ± 0.6	1.6 ± 0.2	<0.005	1.9 ± 1.6	1.8 ± 1.0	0.39
Testosterone (nmol/L)	1.6 ± 1.0	1.05 ± 0.48	<0.0001	1.4 ± 0.8	0.7 ± 0.4	0.005
SHBG (nmol/L)	42.5 ± 39.6	77.5 ± 78.4	0.0003	71.7 ± 62.2	104 ± 80	0.03
Free androgen index (FAI)	4.5 ± 3.9	2.1 ± 1.4	<0.0001	4.1 ± 2.9 ± 4.08	1.3 ± 0.5	<0.0001
CRP (mg/L)	4.4 ± 4.2	2.4 ± 3.9	0.0008	2.8 ± 2.6	2.3 ± 2.34	0.38
AMH (ng/mL)	40 ± 31	18 ± 18	<0.0001	57 ± 14	24 ± 13	<0.0001

BMI—body mass index; HOMA-IR—homeostasis model of assessment—insulin resistance; CRP—C-reactive protein; SHBG—sex hormone-binding globulin.

**Table 2:** Complement proteins in lean and obese patients with polycystic ovary syndrome (PCOS) versus their BMI-matched controls. Data presented as mean ± 1 standard deviation of relative fluorescent units (RFU). Proteins shaded in orange were increased in both cohorts relative to their respective controls; in the final column, *p* values between the nonobese and obese PCOS cohorts that show significant differences following Bonferroni correction for multiple comparisons are indicated by the symbol *.

	LEAN			OBESE			
	**PCOS**	**Control**	***p* Value Nonobese PCOS vs. Controls**	**PCOS**	**Control**	***p* Value Obese PCOS vs. Controls**	***p* Value Nonobese PCOS vs. Obese PCOS**
Properdin	152,592 (42,743)	117,488 (50,041)	0.006	119,125 (26,794)	102,491 (26,374)	0.000003	0.00000001 *
C3b	50,982 (28,296)	46,250 (39,450)	0.6	110,730 (68,743)	105,010 (59972)	0.5	0.000001 *
iC3b	7148 (2127)	5991 (1425)	0.02	14,476 (4563)	11,445 (4188)	0.0000003	1 × 10 ^13^ *
C3	65,878 (26,872)	45,742 (18,189)	0.002	71,028 (25,536)	63,896 (26,822)	0.04	0.33
C3adesArg	152,050 (32,483)	121,110 (45,753)	0.004	168,929 (28,060)	163,048 (30,262)	0.12	0.004
C3a	534 (204)	415 (1010	0.007	1045 (870)	863 (465)	0.06	0.002
C3d	10,427 (3675)	8207 (3261)	0.02	11,905 (3645)	9816 (2890)	0.000004	0.048
C4	119,057 (28,429)	104,245 (28,069)	0.05	121,193 (26,829)	114,603 (25,411)	0.06	0.7
C4A	71,549 (9802)	73,037 (2258)	0.43	116,060 (4320)	116,497 (3708)	0.42	1 × 10 ^14^ *
Factor I	44,861 (6786)	39,960 (7356)	0.01	47,519 (6883)	42304 (6958)	0.00000002	0.06
Factor D	733 (99)	693 (150)	0.24	898 (146)	933 (314)	0.24	0.00000001 *
C2	2878 (319)	2874 (228)	0.96	2694 (386)	2540 (378)	0.002	0.02
Complement factor H-related 5	1454 (399)	1677 (1416)	0.42	1298 (264)	1206 (303)	0.01	0.01
Factor B	30,257 (6541)	28,172 (5791)	0.2	34,162 (5281)	30,078 (5054)	0.00000001	0.006
Factor H	60,898 (9191)	59,289 (6016)	0.43	72,843 (8954)	64,750 (9119)	1 × 10 ^10^	1 × 10 ^10^ *
C5a	14,729 (5811)	11,343 (4953)	0.02	17,983 (7875)	13,106 (6113)	0.000001	0.04
C5b, 6 Complex	521 (62)	502 (62)	0.27	490 (51)	493 (194)	0.87	0.005
C5	7267 (1007)	6881 (1063)	0.16	7432 (1692)	7071 (1088)	0.06	0.61
C1q	35,146 (9269)	35,294 (7886)	0.95	38,519 (7784)	37,027 (7612)	0.14	0.04
C1r	3203 (886)	3740 (3992)	0.48	3414 (1185)	3497 (967)	0.57	0.36
C4b	349 (186)	335 (202)	0.78	846 (913)	889 (632)	0.69	0.004
MBL	12,058 (5835)	13,322 (7385)	0.47	16,835 (7427)	16,298 (79,120)	0.59	0.001 *
DAF (CD55)	15,182 (3220)	13,886 (3339)	0.14	13,958 (2717)	14,424 (2323)	0.17	0.03

## Data Availability

All the data for this study will be made available upon reasonable request to the corresponding author.
